# The interventionalism of medicine: interventional radiology, cardiology, and neuroradiology

**DOI:** 10.1186/1755-7682-2-27

**Published:** 2009-09-09

**Authors:** Shaheen E Lakhan, Anna Kaplan, Cyndi Laird, Yaacov Leiter

**Affiliations:** 1 Global Neuroscience Initiative Foundation, Los Angeles, CA, USA

## Abstract

Interventional medical practitioners are specialists who do minimally invasive procedures instead of surgery or other treatment. Most often, these procedures utilize various imaging and catheterization techniques in order to diagnose and treat vascular issues in the body. Interventionalist techniques, including injecting arteries with dye, visualizing these via x-ray, and opening up blockages, developed from early pioneers' bold and sometimes controversial experiments which aimed to find safer and better ways to treat coronary artery and other atherosclerotic vascular disease. Currently, the major interventional specialties are interventional (or vascular) radiology, interventional cardiology, and endovascular surgical (interventional) neuroradiology. All three are perfecting the use of stents and other procedures to keep diseased arteries open, while also evaluating the application these procedures. The rapid new development of imaging technologies, mechanical devices, and types of treatment, while certainly beneficial to the patient, can also lead to ambiguity regarding specific specialty claims on certain techniques and devices. While these practitioners can be in competition with each other, cooperation and communication are the most advantageous methods to deal with these "turf wars." All of the interventionalists are needed to deliver the best medical care to patients, now and in the future.

## Introduction

In the most general of terms, an interventional medical practitioner is a doctor with a medical specialty who has been trained to do minimally invasive procedures, usually involving blood vessels, which can be done instead of actual surgery. For example, an interventional cardiologist may put a stent, a tiny mesh tube, into a coronary artery to keep it open, whereas a cardiac surgeon may perform a coronary artery bypass in such a case. Similarly, an interventional radiologist (also called a vascular radiologist) may put a stent into a blocked carotid artery to remove the blockage whereas, under similar circumstances, a vascular surgeon may perform an endarterectomy. The benefits of interventionalist techniques thus are in the often reduced recovery time and pain associated with the procedures, due to their less invasive nature.

This field, and its specialties, offers extremely important and beneficial advances in the world of medicine, however, it is not within the scope of this article to describe all the discoveries, nor to exhaustively state the most up-to-date procedures used for treatment. Rather, we aim to highlight the history of these specialties, comment on their current position, and describe some of their future challenges and promises.

## History of Interventionalism

What is now known as interventionalism, in radiology, neuroradiology, or cardiology, began with the investigations and inventions of cardiologists, radiologists and other doctors who were pushing science and medicine into the future.

A necessary foundation for each of these fields was the development of a technology in which the heart and blood vessels were visible on x-ray. This breakthrough came in 1929, when Werner Forssmann, a surgical resident in Germany, put a catheter into his own antecubital vein, advanced it, and took x-rays of himself to prove the catheter was in the right atrium [1-3]. Although his work faced initial rejection, during the 1930's and 40's other doctors recognized his work and began using catheters to measure cardiac output and to introduce drugs into the heart [2,4].

Many additional breakthroughs came throughout the 1950's and 60's. In 1953, Sven-Ivar Seldinger described a percutaneous femoral technique wherein a catheter over a guidewire is advanced through the skin and into the femoral artery; this eventually became the method of choice for arterial vascular access in interventional radiology procedures [4,5]. Shortly after this discovery, Mason Sones, a pediatric cardiologist, accidently found a way to visualize the coronary artery when a bolus of dye was injected into the right coronary artery while he was performing a cardiac catheterization to look at a patient's aortic valve [2,4,6].

Then, in 1964, vascular radiologist Charles Dotter began performing transluminal angioplasty in which he utilized catheters through the brachial artery to open peripheral arteries in the lower extremities [1,2,4,6-8]. Although his work was not widely accepted in the United States until later, one of his associates, Melvin Judkins, worked out his own system of diagnostic imaging using a groin puncture to introduce the catheters [4,6,8] which went on to become the standard approach to angiography in the United States.

Concurrently in Europe, a number of doctors were also working on the same types of procedures. One of these was German cardiologist Andreas Gruentzig. Gruentzig, a student of Dotter's techniques, spent years trying to work out a system to open closed arteries using catheters and inflatable balloons. He performed many experiments on animals, often using devices of his own construction, and eventually applied his techniques to a human. This came in 1976 when he and Richard Myler performed the first human coronary artery angioplasty [2,4,6,8]. It took years of data collection and analysis, and many attempts to perfect the balloons and catheters, but in time it became the accepted practice. It is to a large extent because of his influence in the development of this technique that the field, which was started and progressed through research by both radiologists and cardiologists, was claimed by the interventional cardiologists.

## Interventional Radiology

Interventional radiologists (IR) are board-certified radiologists who specialize in minimally invasive, targeted treatments. More specifically, they use available imaging systems, from x-rays to MRIs, in order to advance a catheter in the body, usually in an artery, to treat the source of disease non-surgically. According to the Society of Interventional Radiology (SIR), "As the inventors of angioplasty and the catheter-delivered stent, IRs pioneered minimally invasive modern medicine [9]." In many ways, interventional radiologists are still at the center of the interventionalist movement.

Charles Dotter is considered the father of angioplasty and interventional radiology. He is remembered as a brilliant innovator and Nobel Prize nominee who authored 300 publications and predicted much of what has eventually come to fruition in the field during the past 40 years. Since Dotter's time, IRs have tried to maintain control of the procedures he and other radiologists helped invent, while also trying to define their specialty [7-9].

The formation of SIR in the 1980's enabled some of this control while also allowing for improved training and activity of IRs. In 1989 SIR established "Special Requirements for Subspecialty Training in Vascular and Interventional Radiology" and by 1993 the Accreditation Council for Graduate Medical Education (ACGME) approved training and started accrediting programs. By 2000, there were some 100 fellowship programs training approximately 200 individuals a year. Examination and certification in interventional radiology commenced in 1995. By 2001 there were 2154 physicians who had obtained the certificate of added qualification in interventional radiology [7].

Specific duties of IR are vast, varied and continuously expanding. Although, for the most part, cardiologists control conventional coronary angiography, as well as stenting and other related procedures in the coronary arteries, IRs are able to image, open and stent the carotid arteries and peripheral arteries, including the renal, popliteal and femoral arteries, among others. They are able to help treat aortic aneurysms and dissections, use percutaneous access to treat issues, and utilize tools, like CT scans, MRI, PET scans and ultrasound to look into many areas of the body to treat and diagnose patients [7,8,10]. IRs also perform numerous nonvascular interventions including procedures of the biliary tree (e.g cholecystostomy and biliary tract decompression), nephrostomy, insertion of peritoneal dialysis catheters, and radiology-guided gastrostomy. In addition, abscess drainage, pleurocentesis, and percutaneous biopsies are part of the everyday job of the IR.

Guiding all of responsibilities of an IR, however, is the obligation to patient well being. In 2000, Gary Becker commented on the future of interventional radiology; he stated that, "Interventional radiology is a discipline with a procedural foundation rooted in diagnostic imaging and dependent on innovation but with a clinical focus that demands our attention and deserves center stage in our practice [8]." He shared Dotter's belief that the interventionalist had a significant role as a clinician that must be preserved as Dotter explained at the American College of Surgery meeting in 1968. Dotter said, "If my fellow angiographers prove unwilling or unable to accept or secure for their patients the clinical responsibilities attendant on transluminal angioplasty, they will become high-priced plumbers facing forfeiture of territorial rights [7]."

As this is a concern among those in the field, interventional radiology training has been expanded with ways to add more clinical time. Today, interventional radiology has three pathways for residents to train in order to become board certified in the subspecialty [11]. The traditional pathway is widely available and involves a clinical internship and four years of diagnostic radiology residency followed by a one-year interventional radiology fellowship. The Diagnostic and Interventional Radiology Enhanced Clinical Training (DIRECT) pathway is new, allowing up to two years of clinical training to count toward the diagnostic radiology certificate and subspecialty vascular interventional radiology certificate. The third, the clinical pathway, provides a broader and more in-depth experience in the clinical diagnosis and care of patients with diseases commonly treated by IRs [11].

Another concern Becker addressed were the turf wars encountered with other specialists wanting to do interventional radiology procedures as the field expands. These thoughts were echoed by many, including Barry Katzen in his 2004 piece on the changes in the ten years since interventional radiology had become an ACGME board-certified specialty. He explained that as soon as less-invasive interventional procedures became accepted, specialists wanted to learn how to do them; specialists including cardiologists and vascular surgeons.

Concerning the future of interventionalist radiology, John A. Kaufman, chair of the 2006 Society of Interventional Radiology meeting has stated that these specialists should focus on people, not diseases; commit to clinical care; and work with many other associates on interventional teams. Accordingly, IRs could be at the forefront of healthcare, with continued cooperative efforts and research.

## Interventional Cardiology

According to The Society for Cardiovascular Angiography and Interventions, "Interventional cardiology is the specialized branch of cardiology that treats coronary artery disease with balloon angioplasty and stenting, therapies that unblock clogged arteries that supply blood to the heart, stop heart attacks and relieve angina, or chest pain [12]." Interventional cardiologists (IC) are also trained to do procedures on cardiac valves and other structures.

Whereas Dotter is the father of interventional radiology, Andreas Gruentzig, who perfected coronary angioplasty, is considered the father of interventional cardiology [4]. He did much of his angioplasty work in the United States at Emory University, where the first controlled trial comparing angioplasty to coronary artery bypass surgery took place. His techniques allowed for visualization of the coronary arteries and better treatment of coronary artery disease. Angioplasty went through many phases in order to combat complications associated with the procedure [6] with the most notable issue being restenosis, or renarrowing, of the coronary arteries. Another problem was and still is the formation of new clots.

Over time, various methods have been used to try and keep the coronary arteries open including the administration of thrombolytic drugs delivered straight into the coronary arteries [6] and the use of stents. Clinical trials in the 1990's showed the benefits of the first metal stents [13] and later, drugs were incorporated into "drug-eluting stents." Notable among these drug-eluting stents is the sirolimus-eluting stent as are the The Rapamycin-Eluting Stent Evaluated at Rotterdam Cardiology Hospital (RESEARCH), Compassionate Use of SES (SECURE), and e-CYPHER stents, of which the latter three are currently undergoing clinical trial investigations [13].

Beyond stenting technology, ICs also have access to CT angiography (CTA) and MR angiography (MRA), techniques which are becoming more and more reliable and useful in the diagnosis of coronary artery disease. It is thought that coronary CTA will no doubt reduce the number of unnecessary invasive angiograms in patients with normal or nonsignificant coronary artery lesions, and may also be used in conjunction with other medical treatment. Practically speaking, CTA will probably have a greater role than MRA, although the technology is advancing faster than studies can evaluate. As both of these technologies become more reliable, however, they become attractive to ICs and IRs alike, further contributing to possible turf wars between the specialists. ICs are also interested in peripheral interventions in the renal and iliac arteries, the carotids, and others [14]. These are areas where they will be in conflict with IRs and interventional neuroradiologists.

Currently, the conditions most treated by ICs are coronary artery disease and its sequelae although there are many other non-coronary interventional procedures that these specialists can perform. Some fellowships require two years instead of one to allow time to learn the less common procedures done for less common cardiac problems. They include, among others, procedures to repair cardiac valves and defects. Percutaneous treatment with balloon dilatation is the treatment of choice for most patients with mitral stenosis, pulmonary stenosis, and congenital aortic stenosis. However, these valvular lesions are only a small percentage of the valvular heart disease spectrum. In these cases, the balloon is used to open the stenosed valve, which is not unlike balloon angioplasty opening a coronary artery. Technological advances may allow percutaneous treatment of other valvular diseases to be done more often in the future [13]. Other interventional procedures include percutaneous coronary sinus repair for the treatment of mitral regurgitation, percutaneous valve insertion for the treatment of calcific aortic valve stenosis, patent foramen ovale closure with multiple devices, transcatheter closure of atrial-septal defects, closure of ventricular-septal defects, left atrial appendage occlusion, placement of percutaneous mechanical assist devices, and alcohol septal ablation for hypertrophic obstructive cardiomyopathy [13].

Looking at the future in 2005, the authors of an article called "New Frontiers in Interventional Cardiology" and published in *Circulation *predicted, "Progress in the areas of drug-eluting stents, detection of vulnerable plaques, percutaneous management of selected patients with stroke and valvular heart disease, angiogenesis and stem cell treatment of congestive heart failure, and increased use of the predictive capacity of genetic markers likely will be pivotal [13]." Other areas that are starting to be explored include therapeutic angiogenesis and myocardial regeneration. Both techniques are experiencing some advancement with gene therapy and stem cell research thought to influence the further progress of both.

## Endovascular Surgical (Interventional) Neuroradiology

Interventional neuroradiology is one of the terms for neurology, neuroradiology and neurosurgery-based practices involving interventional endovascular techniques. This specialty is in the process of redefining and renaming itself, now using the terminology endovascular surgical (EVS) neuroradiology.

According to the ACGME, as of 2009 [15],

Endovascular surgical neuroradiology is a subspecialty that uses minimally invasive catheter-based technology, radiologic imaging, and clinical expertise to diagnose and treat diseases of the central nervous system, head, neck, and spine. The unique clinical and invasive nature of this subspecialty requires special training and skills.

While there are many diagnostic and therapeutic procedures done by EVS neuroradiologists, most of them involve arterial imaging and treating carotid artery stenosis [16]. Major tasks executed by EVS neuroradiologists include carotid stenting, cerebral arteriograms, procedures for intracranial aneurysms and arteriovenous malformations, as well as treatments for some head or neck disorders and some cancers. Interestingly, however, The National Inpatient Sample of hospitalized patients indicated that in 2001 there were about 151,000 patients with carotid stenosis who underwent endovascular procedures and over 133,000 cerebral arteriograms performed, as opposed to just under 21,000 procedures for intracranial aneurysms and 240 for arteriovenous malformations [16]. Thus, despite the fact that there are many other procedures an EVS neuroradiologist can do most of the time he or she will be performing vascular studies and dealing with the carotid arteries.

There are many ongoing studies regarding diagnosis and treatment of carotid artery stenosis [17]. The original treatment and "gold standard" has been surgical carotid endarterectomy (CEA) performed by vascular surgeons worldwide. This procedure has been compared to carotid angioplasty with and without stenting in several studies, many of which are ongoing. At first, angioplasty and carotid artery stenting (CAS) were used for patients who were not candidates for the more invasive surgical procedure and the Carotid and Vertebral Artery Transluminal Angioplasty Study (CAVATAS), reported in 2001, indicated benefits for CAS [18]. In 2003, the Study of Angioplasty with Protection in Patients at High Risk for Endarterectomy (SAPPHIRE) compared carotid angioplasty with stent placement to carotid endarterectomy in 307 high surgical risk patients with symptomatic or asymptomatic stenosis [19]. At 1 year, the ipsilateral stroke rates were 4% and 5%, respectively, for carotid angioplasty/stenting versus endarterectomy. Overall, 1-year mortality was 7% for the endovascular patients and 13% for the surgical cohort. Moreover, 3-year follow-up data demonstrated no significant difference in long-term outcomes between the two cohorts [20].

The multicenter Carotid Revascularization Endarterectomy versus Stent Trial (CREST), which is still underway, is comparing the efficacy of CEA and CAS in symptomatic patients. Its lead-in phase was designed to make sure the surgeons and interventionalists in both arms provided the best available therapy. Preliminary data reported in 2003 showed that "periprocedural morbidity and mortality rates for CAS performed by experienced interventionalists are comparable to that reported from large randomized trials of CEA and comparable or lower than that previously reported [21]."

EVS neuroradiologists stenting the carotid arteries are trying stents with new protective devices to capture dislodged material at the distal end. These devices should help reduce embolic events and neurologic damage after the procedure [16,21]. Percutaneous angioplasty and stenting are also being used for intracranial stenosis and extracranial vertebrobasilar stenosis [16].

There have been other procedures revolutionizing treatment of intracranial vascular abnormalities. EVS neuroradiology has made great strides in treating intracranial aneurysms. The Guglielmi detachable coil was the first device approved to embolize intracranial aneurysms, and new devices using new materials are being tested. The coils are also being used to treat both ruptured and intact aneurysms, and results obtained are better than surgical clipping [16]. CNS arteriovenous malformations can also be treated with a variety of devices. New imaging systems and new technical advances continue to make what were once untreatable vascular disorders treatable conditions.

## The Current State and Future of Interventional and Endovascular Therapies

The future for interventional therapists continues to evolve. These doctors are continuously improving upon minimally invasive procedures to treat common medical conditions. Even with improved medical therapy for atherosclerotic vascular disease, which may negate the need for some interventional procedures, the population is aging and there will be enormous numbers of patients needing care for coronary artery disease and carotid atherosclerosis, along with other less common conditions.

IRs already diagnose cancer by needle biopsy and deliver treatment directly to cancer sites, whether radiation or radiofrequency ablation or another technique [22]. In the future, it may be gene therapy, for example, that will help revolutionize treatment. EVS neuroradiologists are also treating head, neck and even brain tumors with arterial embolization and intra-arterial chemotherapy. They, too, look to a future with gene therapy and stem cells [16]. ICs are already delivering progenitor cells such as bone marrow stromal cells into the heart to try and repair damaged myocardium. It is not possible yet to say which therapies will emerge as the most useful, but doubtless research by interventionalists will continue to play a role in these areas.

Along with the many advances these new technologies promise, however, they also provide a basis of contention between specialties. As mentioned previously, some techniques, such as carotid stenting, have already provided a ground for turf wars, with further interest coming from vascular surgeons, nephrologists and others who are already learning interventional techniques [7].

Perhaps the most positive outcome is that they get together and cooperate, as they are doing in the Carotid Revascularization by Endarterectomy versus Stenting Trial (CREST) study. This large trial comparing carotid endarterectomies to carotid stent placements finds IRs, neuroradiologists and ICs, along with vascular and neurosurgeons working together to look closely at outcomes with traditional surgery, as opposed to stenting [7]. In the case of CREST, the most recognized interventionalists are working together, but there are other specialists interested in learning many of these techniques [7,8].

As another example, a group worked together to write guidelines for cerebrovascular diagnosis and treatment. A position paper, published in *Neurology *in 2005, called "Training, competency, and credentialing standards for diagnostic cervicocerebral angiography, carotid stenting, and cerebrovascular intervention: A Joint Statement from the American Academy of Neurology, the American Association of Neurological Surgeons, the American Society of Interventional and Therapeutic Neuroradiology, the American Society of Neuroradiology, the Congress of Neurological Surgeons, the AANS/CNS Cerebrovascular Section, and the Society of Interventional Radiology" brought many practitioners together to work on training guidelines. This document also states, "These organizations represent all clinical medical specialties with formal accredited ACGME-approved training in the cervicocerebral vasculature and associated neurological pathophysiology. The executive committees and governing bodies of each organization have approved this document [23]." The authors claim that EVS neuroradiologists are the group best suited for these procedures, but details the specifics of training other specialists enough to qualify.

As mentioned above, as CT and MRI coronary angiograms improve and gain acceptance, interest in their application will also increase. IRs and ICs have commented on this reality themselves, each side making the case for their own specialty to have control [24]. Mention was made of the "The Manhattan Project" named after the World War II atomic weapons development program. This project, initiated by the Society of Chairmen in Academic Radiology Departments, was to provide special training, especially to radiologists, in cardiac and vascular imaging. Although the claim from coordinator and founder of the project Dieter Enzmann, a radiologist, was that " [this program] is nothing more than an attempt on radiology's part to identify sites around the country that could train other radiologists and in fact other cardiologists in cardiac imaging: MRI and CT [24]."

When it came to drafting a statement about CT and MR coronary angiograms to be published in *Circulation *in 2008, however, the cardiologists worked with cardiovascular surgeons to write these guidelines, whereas input from IRs was unsolicited [25].

In the end, whether or not they will work together cooperatively or each try to carve out their own turf will probably vary from place to place. What happens at a teaching hospital will be different from what happens at a small hospital away from a major metropolis. If all the interventionalists remember to put the patients' best interests first, however, the continuing advancement and future developments of these fields should follow with positive and progressive outcomes.

## Abbreviations

AANS: American Association of Neurological Surgeons; ACGME: Accreditation Council for Graduate Medical Education; CAS: carotid artery stenting; CAVATAS: Carotid and Vertebral Artery Transluminal Angioplasty Study; CEA: carotid endarterectomy; CNS: central nervous system; CREST: Carotid Revascularization by Endarterectomy versus Stenting Trial; CT: computed tomography; CTA: computed tomography angiography; DIRECT: Diagnostic and Interventional Radiology Enhanced Clinical Training; EVS: endovascular surgical; MR: magnetic resonance; IC: interventional cardiologist; IR: interventional radiologist; MRA: magnetic resonance angiography; MRI: magnetic resonance imaging; PET: positron emission tomography; RESEARCH: Rapamycin Eluting Stent Evaluated at Rotterdam Cardiology Hospital; SAPPHIRE: Study of Angioplasty with Protection in Patients at High Risk for Endarterectomy; SECURE: Compassionate Use of SES; SIR: Society of Interventional Radiology; TIPS: transjugular intrahepatic portosystemic shunt.

## Competing interests

The authors declare that they have no competing interests.

## Authors' contributions

All authors participated in the preparation of the manuscript, and read and approved the final manuscript.

## References

[B1] Neiman HL, Lyons J, Ascher E, Ascher E (2004). Chapter 5: Fundamentals of Angiography. Vascular Surgery.

[B2] History of Angioplasty. http://www.ptca.org/history_timeline.html.

[B3] Biographical sketch of Werener Forssmann. http://www.ptca.org/archive/bios/forssmann.html.

[B4] Ryan TJ (2002). Medicine over five decades: The coronary angiogram and its seminal contributions to cardiovascular medicine over five decades. Circulation.

[B5] Greitz T (1999). Sven-Ivar Seldinger. American Journal of Neuroradiology.

[B6] King SB (1996). Angioplasty from bench to bedside to bench. Circulation.

[B7] Becker GJ (2001). The future of interventional radiology. Radiology.

[B8] Rosch MD, Keller FS, Kaufman JA (2003). J Vasc Interv Radiol. The birth, early years, and future of interventional radiology.

[B9] What Are Interventional Radiologists?. http://www.sirweb.org/patients.

[B10] Athanasoulis CA (2001). Vascular radiology: Looking into the past to learn about the future. Radiology.

[B11] IR Pathway Options. http://www.sirweb.org/fellows-residents-students/pathway-options.shtml.

[B12] Frequently Asked Questions: 30th Anniversary of Interventional Cardiology. http://www.scai.org/pr.aspx?PAGE_ID=5187.

[B13] Sousa JE, Costa MA, Tuzcu EM, Yadav JS, Ellis S (2005). New frontiers in interventional cardiology. Circulation.

[B14] Herrmann HC (2005). Interventional Cardiology: Percutaneous Noncoronary Intervention.

[B15] Requirements for Fellowship Education in Endovascular Surgical Neuroradiology. http://www.acgme.org/acWebsite/downloads/RRC_progReq/422pr403.pdf.

[B16] Qureshi AI (2004). Ten years of advances in neuroendovascular procedures. J Endovasc Ther.

[B17] Kirmani JF, Janjua N, Kawi AL, Ahmed S, Khatri I, Ebrahimi A, Divani AA, Quershi AI (2005). Therapeutic advances in interventional neurology. NeuroRx.

[B18] CAVATAS investigators (2001). Endovascular versus surgical treatment in patients with carotid stenosis in the Carotid and Vertebral Artery Transluminal Angioplasty Study (CAVATAS): a randomised trial. Lancet.

[B19] Yadav JS, Wholey MH, Kuntz RE, Fayad P, Katzen BT, Mishkel GJ, Bajwa TK, Whitlow P, Strickman NE, Jaff MR, Popma JJ, Snead DB, Cutlip DE, Firth BG, Ouriel K, Stenting and Angioplasty with Protection in Patients at High Risk for Endarterectomy Investigators (2004). Protected carotid-artery stenting versus endarterectomy in high-risk patients. N Engl J Med.

[B20] Gurm HS, Yadav JS, Fayad P, Katzen BT, Mishkel GJ, Bajwa TK, Ansel G, Strickman NE, Wang H, Cohen SA, Massaro JM, Cutlip DE, SAPPHIRE Investigators (2008). Long-term results of carotid stenting versus endarterectomy in high-risk patients. N Engl J Med.

[B21] Hobson RW, CREST Executive Committee (2003). Carotid stenting in the CREST lead-in phase: Periprocedural stroke, myocardial infarction, and death rates. Stroke.

[B22] Georgy BA (2008). Metastatic spinal lesions: State-of-the-art treatment options and future trends. Am J Neuroradiol.

[B23] Connors JJ, Sacks D, Furlan AJ, Selman WR, Russell EJ, Stieg PE, Hadley MN, Wojak JC, Koroshetz WJ, Heros RC, Strother CM, Duckwiler GR, Durham JD, Tomsick TO, Rosenwasser RH, McDougall CG, Haughton VM, Derdeyn CP, Wechsler LR, Hudgins PA, Alberts MJ, Raabe RD, Gomez CR, Cawley CM, Krol KL, Futrell N, Hauser RA, Frank JI, American Academy of Neurology; American Association of Neurological Surgeons; American Society of Interventional and Therapeutic Neuroradiology; American Society of Neuroradiology; Congress of Neurological Surgeons; AANS/CNS Cerebrovascular Section; Society of Interventional Radiology; NeuroVascular Coalition Writing Group (2005). Training, competency, and credentialing standards for diagnostic cervicocerebral angiography, carotid stenting, and cerebrovascular intervention: A Joint Statement from the American Academy of Neurology, the American Association of Neurological Surgeons, the American Society of Interventional and Therapeutic Neuroradiology, the American Society of Neuroradiology, the Congress of Neurological Surgeons, the AANS/CNS Cerebrovascular Section, and the Society of Interventional Radiology. Neurology.

[B24] Cardiologists and radiologists gear up for CT angiography turf war. http://www.theheart.org/article/373071.do.

[B25] Bluemke DA, Achenbach S, Budoff M, Gerber TC, Gersh B, Hillis LD, Hundley WG, Manning WJ, Printz BF, Stuber M, Woodard PK (2008). Noninvasive coronary artery imaging. Magnetic resonance angiography and multidetector computed tomography angiography. A scientific statement from the American Heart Association Committee on Cardiovascular Imaging and Intervention of the Council on Cardiovascular Radiology and Intervention, and the Councils on Clinical Cardiology and Cardiovascular Disease in the Young. Circulation.

